# How much global climate adaptation finance is targeting the health sector?

**DOI:** 10.1093/eurpub/ckac129.146

**Published:** 2022-10-25

**Authors:** T Alcayna, D O'Donnell

**Affiliations:** Red Cross Climate Centre, The Hague, Netherlands

## Abstract

Climate change seriously threatens health and wellbeing with projected health burdens estimated to cost USD 2-4 billion a year by 2030. Adaptation in the health sector is critical to keep pace with the ongoing consequences of the climate crisis and the impacts projected to occur in the next decade and beyond. Yet estimates by the WHO indicate that climate finance targeting the health sector to date is extremely low: less than 0.5% of multilateral climate adaptation funding has targeted the health sector. In this study, we trace and quantify the amount of adaptation financing targeting the health sector from both multilateral and bilateral sources using publicly available information on the OECD-DAC database and Climate Funds Update. We find that between 2009-2019 only 0.39% of multilateral and bilateral climate adaptation funding targeted health-related efforts specifically. Despite the relatively higher number of health-related projects in Sub-Saharan Africa compared to other regions, a smaller amount of funding is allocated per project compared to other regions. Regional variations in funding are concerning as the countries with the most vulnerability to the climate crisis coincide with regions getting the least amount of funding per project. There is a significant gap in globally financed adaptation efforts in the health sector. Swift and committed remediation is needed to minimise the spiralling risk of high negative health outcomes.

**Key messages:**

• Between 2009-2019 only 0.39% of multilateral and bilateral climate adaptation funding targeted health-related efforts specifically.

• The countries with the most vulnerability to the climate crisis coincide with regions getting the least amount of funding per health project.

